# Vision–language foundation model for echocardiogram interpretation

**DOI:** 10.1038/s41591-024-02959-y

**Published:** 2024-04-30

**Authors:** Matthew Christensen, Milos Vukadinovic, Neal Yuan, David Ouyang

**Affiliations:** 1 Department of Cardiology, Smidt Heart Institute, Cedars-Sinai Medical Center, Los Angeles, CA USA; 2 Department of Bioengineering, University of California Los Angeles, Los Angeles, CA USA; 3 Department of Medicine, University of California San Francisco, San Francisco, CA USA; 4 Division of Cardiology, San Francisco Veterans Affairs Medical Center, San Francisco, CA USA; 5 Division of Artificial Intelligence in Medicine, Cedars-Sinai Medical Center, Los Angeles, CA USA

**Keywords:** Cardiovascular diseases, Computational platforms and environments

## Abstract

The development of robust artificial intelligence models for echocardiography has been limited by the availability of annotated clinical data. Here, to address this challenge and improve the performance of cardiac imaging models, we developed EchoCLIP, a vision–language foundation model for echocardiography, that learns the relationship between cardiac ultrasound images and the interpretations of expert cardiologists across a wide range of patients and indications for imaging. After training on 1,032,975 cardiac ultrasound videos and corresponding expert text, EchoCLIP performs well on a diverse range of benchmarks for cardiac image interpretation, despite not having been explicitly trained for individual interpretation tasks. EchoCLIP can assess cardiac function (mean absolute error of 7.1% when predicting left ventricular ejection fraction in an external validation dataset) and identify implanted intracardiac devices (area under the curve (AUC) of 0.84, 0.92 and 0.97 for pacemakers, percutaneous mitral valve repair and artificial aortic valves, respectively). We also developed a long-context variant (EchoCLIP-R) using a custom tokenizer based on common echocardiography concepts. EchoCLIP-R accurately identified unique patients across multiple videos (AUC of 0.86), identified clinical transitions such as heart transplants (AUC of 0.79) and cardiac surgery (AUC 0.77) and enabled robust image-to-text search (mean cross-modal retrieval rank in the top 1% of candidate text reports). These capabilities represent a substantial step toward understanding and applying foundation models in cardiovascular imaging for preliminary interpretation of echocardiographic findings.

## Abstract (web summary)

A vision–language foundation model, trained on a dataset of more than 1 million echocardiogram video–text pairs, is able to assess various cardiac structural and functional parameters despite not having been directly trained on any specific image interpretation task.

## Main

Echocardiography, or cardiac ultrasound, is the most common, noninvasive method of evaluating heart function and identifying heart disease. Echocardiography routinely guides clinical cardiology decision-making^1–3^ and is used for disease diagnosis, risk stratification and assessment of treatment response^1,4^. Recent work has used artificial intelligence (AI) to improve the accuracy of echocardiographic measurements^5–7^ and disease diagnoses^8–10^; however, these AI approaches focus on narrow individual tasks that require specific training for each task and do not use vision–language foundation models^11^.

Recent advances in AI have leveraged representation learning on large image and text datasets to develop vision–language foundation models that generalize beyond narrow sets of predefined tasks^12,13^. These models learn to encode images and text into compact representations that can then be used to perform a wide variety of separate prediction tasks for which the model was never specifically trained (‘zero-shot’ tasks). Given the broad range of data used to train these models, the performance of foundation models are often more robust than with conventional convolutional neural networks^14,15^. In biomedical applications, foundation models have been developed to organize biological^16–18^ and medical^19^ datasets, including modality-specific models for chest X-rays, retinal imaging, wearable waveforms and pathology images^20–25^. Training of foundation models on medical imaging has been bottlenecked by dataset size and is often limited to publicly available data that may not represent the range of disease severities and possible presentations. While text information might be imprecise, clinician evaluations of medical imaging provide an information-rich distillation of complex data.

In this work, we introduce EchoCLIP, a foundation model for echocardiography trained on a dataset of 1,032,975 echocardiogram videos sourced from over a decade of clinical imaging. We developed a method for substantially compressing echocardiography reports, simplifying the matching of clinical text assessments to images to focus on important clinical concepts. To assess the model’s performance, we tested the model’s ability to assess cardiac function, pulmonary artery pressure (PAP) and chamber size, as well as identify common intracardiac devices in both held-out internal test cohorts as well as external test cohorts. By using the model to compare pairs of echocardiogram studies, we can assess the model’s ability to identify unique patients across time, identify clinically important changes in disease state and retrieve relevant clinical text for given images. Finally, we propose a new vision–language model interpretation approach based on matching relevant text with important regions of interest in images.

## Results

EchoCLIP is an echocardiography vision–language model trained with 1,032,975 video–text pairs derived from 224,685 echocardiography studies across 99,870 patients across a decade of clinical care (Table 1). In a self-supervised approach, EchoCLIP is trained on pairs of echocardiogram images (randomly sampled from video frames) and associated clinical report text without direct labeling of clinical interpretations or measurements. The EchoCLIP model uses a ConvNeXt-Base^26^ image encoder and a Byte-Pair Encoding text tokenizer^27^. The text encoder architecture is a decoder-only transformer identical to the architecture used by the original CLIP paper^23^ and has an input context length of 77 tokens. Despite not being directly trained on specific interpretation tasks, EchoCLIP can accurately identify implanted devices as well as assess cardiac form and function (Table 2). To assess the importance of pretraining and architecture^28^, different architectures and dataset configurations were compared (Supplementary Table 1).

**Table 1:** Clinical characteristics of Cedars-Sinai Medical Center study cohort, reported per echocardiography study

	Total	Training	Validation	Test
*n*	224,685	195,082	8,119	21,484
Age (mean (s.d.))	66.26 (16.74)	66.3 (16.7)	65.8 (17.0)	65.7 (16.9)
Female = true (%)	96,451 (42.9)	83,700 (42.9)	3,363 (41.4)	9,388 (43.7)
Race (%)				
Native American	526 (0.2)	456 (0.2)	23 (0.3)	47 (0.2)
Asian	16,601 (7.5)	14,450 (7.5)	555 (6.9)	1,596 (7.5)
Black	29,546 (13.3)	25,624 (13.3)	1,104 (13.8)	2,818 (13.3)
Hispanic	22,424 (10.1)	19,394 (10.0)	842 (10.5)	2,188 (10.3)
Non-Hispanic white	133,399 (60.0)	116,044 (60.1)	4,699 (58.6)	12,656 (59.6)
Other	15,376 (6.9)	13,243 (6.9)	612 (7.6)	1,521 (7.2)
Pacific Islander	767 (0.3)	688 (0.4)	36 (0.4)	43 (0.2)
Unknown	3,700 (1.7)	3,182 (1.6)	149 (1.9)	369 (1.7)
AF	46,994 (20.9)	41,214 (21.1)	1,633 (20.1)	4,147 (19.3)
HF	75,358 (33.5)	65,802 (33.7)	2,764 (34.0)	6,792 (31.6)
HTN	90,738 (40.4)	79,229 (40.6)	3,250 (40.0)	8,259 (38.4)
CVA/TIA/TE	38,283 (17.0)	33,475 (17.2)	1,378 (17.0)	3,430 (16.0)
MI	14,983 (6.7)	13,120 (6.7)	514 (6.3)	1,349 (6.3)
CAD	55,659 (24.8)	48,840 (25.0)	2,040 (25.1)	4,779 (22.2)
PAD	23,369 (10.4)	20,475 (10.5)	838 (10.3)	2,056 (9.6)
DM	37,900 (16.9)	33,226 (17.0)	1,351 (16.6)	3,323 (15.5)
CKD	40,947 (18.2)	35,960 (18.4)	1,482 (18.3)	3,505 (16.3)
Previous smoker	7,632 (3.4)	6,593 (3.4)	256 (3.2)	783 (3.6)

AF, atrial fibrillation; HF, heart failure; HTN, hypertension; CVA, cerebrovascular accident; TIA, transient ischemic attack; TE, thromboembolism; MI, myocardial infarction; CAD, coronary artery disease; PAD, pulmonary artery disease; DM, diabetes mellitus; CKD, chronic kidney disease.

**Table 2:** Main performance metrics

	Image encoder	Tokenizer	MCMRR	LVEF, MAE	PAP, MAE	TAVR, AUC	MitraClip, AUC	Pacemaker, AUC
CLIP	ViT-B-32	CLIP BPE	10,743.0	20.8 (20.7–20.8)	16.8 (16.8–16.9)	0.46 (0.46–0.47)	0.53 (0.52–0.54)	0.51 (0.51–0.52)
EchoCLIP	ConvNeXt	CLIP BPE	571.3	**8.4 (8.3**–**8.4)**	**10.8 (10.8**–**10.9)**	**0.92 (0.91**–**0.92)**	**0.97 (0.97**–**0.97)**	**0.84 (0.84**–**0.84)**
EchoCLIP-R (full-report prompts)	ConvNeXt	Template tokenizer	**206.1**	10.9 (10.9–11.0)	13.2 (13.1–13.2)	0.85 (0.85–0.86)	0.95 (0.94–0.95)	0.77 (0.77–0.78)
EchoCLIP-R (base)	ConvNeXt	Template tokenizer	**206.1**	16.9 (16.8–17.0)	17.5 (17.4–17.5)	0.52 (0.51–0.52)	0.81 (0.81–0.82)	0.66 (0.65–0.66)

Retrieval ranks are out of 21,484 candidates. Performance of the best-performing model for each metric is bolded. Ranges in parentheses indicate 95% CI bootstrapped with 1,000 random samples. MCMRR, mean cross-modal retrieval rank; BPE, Byte-Pair Encoding.

To fit an entire echocardiography report into the text encoder, a domain-specific echocardiography text tokenization format succinctly summarizing common cardiovascular concepts was developed. The model variant trained with this tokenization format, EchoCLIP-R, is capable of retrieving relevant clinical text from images and characterizes clinical changes over time. We also introduce a saliency mapping approach based on cosine similarity, PromptCAM, to show that EchoCLIP prioritizes important image features relevant to the associated text. This approach identifies clinically relevant regions of interest in echocardiography images based on prompted clinical text.

### Echocardiogram interpretation without supervised learning

Without fine-tuning or task-specific training, we evaluated EchoCLIP’s performance on a wide range of benchmark classification tasks in our internal held-out test set. EchoCLIP can accurately identify intracardiac devices, including percutaneous mitral valve repair with an AUC of 0.97 (95% CI 0.97–0.98), transvenous aortic valve replacement (TAVR) with an AUC of 0.92 (95% CI 0.91–0.92) and pacemaker/defibrillator leads with an AUC of 0.84 (95% CI 0.84–0.85). EchoCLIP can also detect changes from a healthy cardiac chamber size, including severe dilation of the right ventricle with an AUC of 0.92 (95% CI 0.91–0.92), right atrium with an AUC of 0.97 (95% CI 0.97–0.98), left ventricle with an AUC of 0.92 (95% CI 0.92–0.93) and left atrium with an AUC of 0.91 (95% CI 0.90–0.92). Last, EchoCLIP can assess for tamponade (AUC 0.96, 95% CI 0.94–0.98) and severe left ventricular hypertrophy (AUC 0.82, 95% CI 0.81–0.83). The sensitivity and specificity for each task are described in Extended Data Table 1. Performance was similar across key subsets stratified by age, sex and image quality (Supplementary Table 2).

### External validation of cardiac function and pressure assessment

**Fig. 1:**
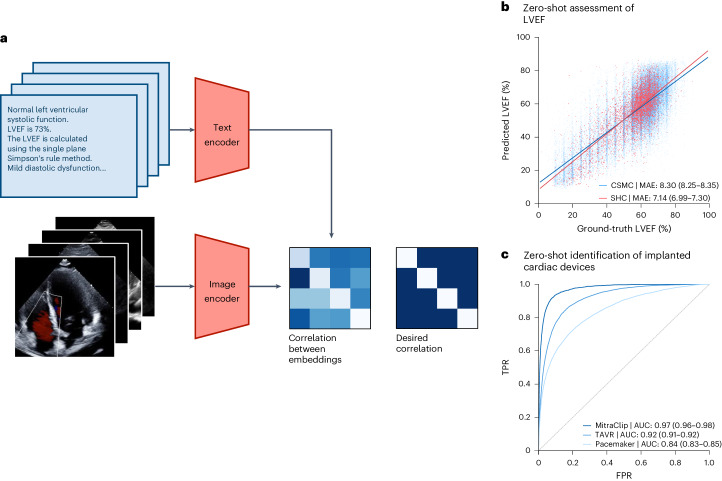
EchoCLIP workflow. **a**, EchoCLIP is a foundation model trained on more than 1 million echocardiogram videos across 11 years. It is composed of an image encoder for processing echocardiogram video frames and a text encoder for processing the corresponding physician interpretations. These two encoders project the images and interpretations onto a joint embedding space. **b**, Scatter-plot of zero-shot prediction versus label of left ventricular ejection fraction (LVEF) in held-out test dataset from Cedars-Sinai Medical Center (CSMC; blue, *n* = 100,994) and Stanford Healthcare (SHC; red, *n* = 5,000). **c**, AUC performance for various implanted intracardiac devices, including MitraClip, TAVR valves and implanted pacemaker/defibrillator on held-out test dataset from Cedars-Sinai Medical Center. FPR, false positive rate; TPR, true positive rate.

**Fig. 2:**
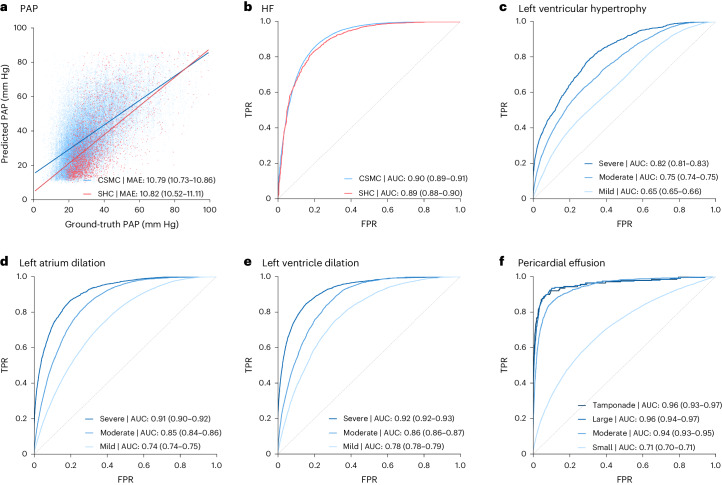
Zero-shot model performance on held-out test apical-four-chamber videos. **a**, Estimation of pulmonary artery pressure (PAP). **b**, Heart failure (HF) with reduced ejection fraction. **c**, Assessment of left ventricular hypertrophy at various degrees of severity (mild, moderate and severe). **d**, Left atrial dilation at various degrees of severity (mild, moderate and severe). **e**, Left ventricular dilation at various degrees of severity (mild, moderate and severe). **f**, Assessment of pericardial effusion size (small, moderate and large) as well as presence of tamponade physiology. Data are from the Cedars-Sinai Medical Center (CSMC; blue, *n* = 100,994) and Stanford Healthcare (SHC; red, *n* = 5,000). FPR, false positive rate; TPR, true positive rate.

We further evaluated EchoCLIP’s performance on quantitative tasks, including evaluation of left ventricular ejection fraction (LVEF) and PAP. EchoCLIP predicts LVEF on the held-out internal test dataset with a mean absolute error (MAE) of 8.4% and an MAE of 7.1% on an external test set of videos from the EchoNet-Dynamic dataset from Stanford Healthcare (Fig. 1). At key clinical LVEF thresholds, EchoCLIP achieves an AUC of 0.89–0.90 for an LVEF threshold of 50%, 0.93–0.94 for an LVEF threshold of 40% and 0.95–0.97 for an LVEF threshold of 30% (Supplementary Table 3 and Supplementary Fig. 1). Furthermore, EchoCLIP predicts estimated PAP with an MAE of 10.8 mm Hg on the internal test dataset and an MAE of 10.8 on the external test dataset (Fig. 2).

### Mapping clinical text to echocardiogram images

Given the long length of an echocardiography report, we developed EchoCLIP-R, a domain-specific text encoder that succinctly summarized common cardiovascular concepts into fewer tokens and was able to summarize a whole report during training. A long-context EchoCLIP-R model was optimized for retrieval during training. Given a representative image from the held-out test cohort, EchoCLIP-R ranks the matching clinical report on average 209th out of 21,484 candidates (top 1% retrieval). The correct report is present in the top ten reports 33.3% of the time. Going from text to image, the average rank of the matching video is 203 out of 21,484 and the correct video is present in the top ten ranked videos 34.3% of the time. For all language models, the choice of text prompts impacts model performance and we found EchoCLIP to be easier to generate focused prompts compared to EchoCLIP-R given the larger context for in-domain prompts (Table 2). A workflow for automated preliminary assessment of echocardiogram studies by ensembling assessments across videos is shown in the Supplementary Video.

### Detection of clinical differences between videos

**Fig. 3:**
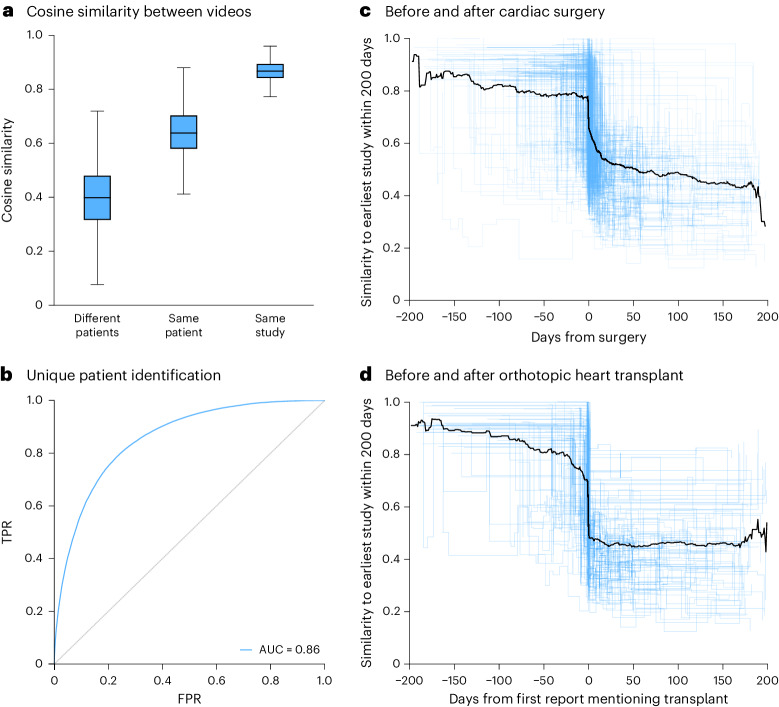
Assessment of clinical similarity. **a**, Average cosine similarity between embeddings from different patients, same patients at different times and same patients at the same time point. Center lines indicate the median, boxes span from the first to the third quartile and whiskers stretch 1.5 × the interquartile range (*n* = 100,994). **b**, AUC for predicting whether the images come from the same patient when compared to another image (*n* = 100,994). **c**,**d**, Trajectory of individual patients by cosine similarity (*n* = 2,959). Each line represents an individual patient with time from major clinical event on the *x* axis and cosine similarity versus first study on the *y* axis. Patients either had major cardiac surgery (**c**) or heart transplant (**d**), with cosine similarity calculated at the study level and pairwise compared for all videos in each study. Data are from the Cedars-Sinai Medical Center. FPR, false positive rate; TPR, true positive rate.

The ability to measure the similarity between pairs of echocardiograms can also be used to identify a unique patient across multiple studies (a difficult task for human clinicians) as well as identify clinical changes over time. Comparing the cosine similarity between EchoCLIP-R embeddings of different echocardiography studies can help in challenging clinical scenarios. Pairs of EchoCLIP-R embeddings of echocardiograms are, on average, least similar if they come from two different patients (mean cosine similarity 0.40, 95% CI 0.39–0.41), more similar if they come from the same patient but were acquired on different dates (mean cosine similarity 0.64, 95% CI 0.64–0.65) and most similar if they come from the same patient and were acquired on the same day (mean cosine similarity 0.87, 95% CI 0.86–0.87). This comparison results in an AUC of 0.86 (95% CI 0.85–0.87) in identifying the same patients across different videos. Furthermore, the cosine similarity between videos can also be used to distinguish when there was a substantive clinical change. Echocardiograms acquired before cardiac surgeries and orthotopic heart transplants tend to be similar to one another, while being substantially less similar to echocardiograms acquired after such procedures (Fig. 3). This dropoff in embedding similarity is sufficient to predict whether an echocardiogram occurs before or after cardiac surgery with an AUC of 0.77 (95% CI 0.75–0.79) and before or after heart transplant with an AUC of 0.79 (95% CI 0.76–0.82). Additionally, we show that the difference in reported LVEF between different studies from the same patient is correlated with the cosine similarity between videos, suggesting that EchoCLIP-R embeddings can be used to identify clinically relevant serial changes (Supplementary Fig. 2).

### Interpretation studies

**Fig. 4:**
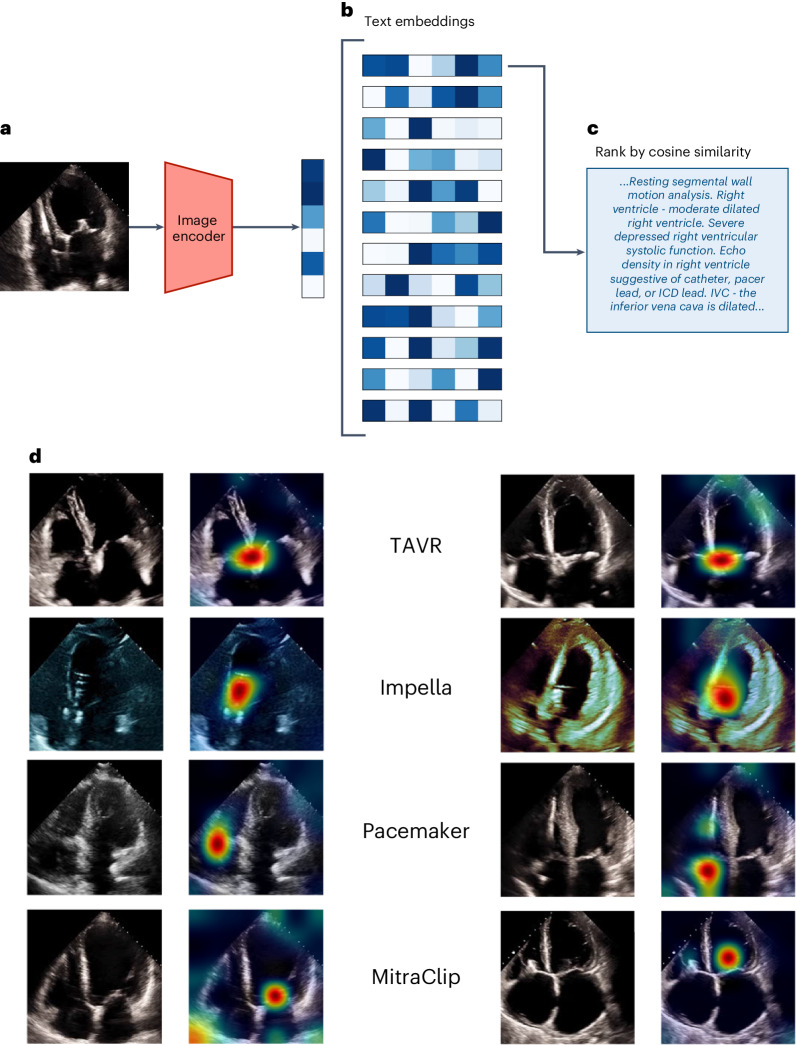
Image-to-text semantic search. **a**, The query image is first embedded using EchoCLIP-R’s image encoder. **b**, Then, the similarities between this query embedding and the embeddings of all 21,484 unique text reports in the test set are computed. **c**, The reports are ranked by their similarity to the query image embedding and the report with the highest similarity is retrieved. **d**, Corresponding pairs of input frames and PromptCAM visualization of the indicated intracardiac devices in the text report label (color intensity ranging from red for most important to green for less important and no color for not important).

To further interrogate EchoCLIP’s understanding of cardiovascular disease, we utilized two interpretability frameworks. First, we developed a modified class activation mapping method (PromptCAM) for multimodal models that pairs textual prompts with imaging features. PromptCAM identifies regions of interest in the image that maximize the cosine similarity with the text prompts. Despite not seeking to minimize the loss of direct text labels in training, PromptCAM highlights the learned associations of EchoCLIP for subconcepts such as ‘TAVR’, ‘Impella’, ‘Pacemaker’ or ‘Mitraclip’ (Fig. 4). Secondarily, we applied Uniform Manifold Approximation and Projection (UMAP) on the embeddings from the EchoCLIP image encoder and observed numerous clusters associated with different cardiovascular diseases, disease states and measurements (Supplementary Fig. 3).

## Discussion

Our results suggest that large datasets of echocardiography studies and expert adjudicated interpretations can serve as the basis for training medical foundation models. Our echocardiography foundation model was able to successfully complete multiple benchmarks of zero-shot prediction tasks without task-specific training or fine-tuning. By training EchoCLIP with data from one healthcare system and testing its performance on data from an entirely separate external healthcare system, we were able to evaluate EchoCLIP’s generalizability and robustness to domain shift. Additionally, EchoCLIP-R displays an ability to perform tasks that human clinicians struggle with or find laborious, such as identifying the same patient across different imaging studies and characterizing clinically important changes over time. Finally, we introduce a multimodal interpretability approach using cosine similarity-based saliency to demonstrate that EchoCLIP has learned semantically meaningful imaging features of both common and rare cardiovascular concepts based on text prompting.

A key bottleneck in training medical foundation models is the limited availability of medical training data. Previous echocardiography AI models were trained with a maximum of 150,000 echocardiogram videos^29^ and most frequently trained with only hundreds or thousands of examples^7,10,30–32^. By leveraging large clinical reporting databases, our approach minimizes the tedious manual labeling and organization required for supervised learning tasks and allows EchoCLIP to be trained on over 1 million echocardiography videos. In its most basic form, the task of mapping images to corresponding text interpretations is the clinical task of medical image interpretation that cardiologists do daily. EchoCLIP represents an opportunity to automate many interpretation tasks simultaneously without the need for individually tuned specialist models, which can ultimately lead to automated preliminary echocardiography interpretation in underserved populations or during emergent situations. A self-supervised vision–language foundation model trained on the diverse range of physiologies seen in a high-volume echocardiography laboratory can learn from greater amounts of data than purely supervised models and may be able to gain a much more generally applicable understanding of the human heart, its function and its structure.

While EchoCLIP is not the first instance of a foundation model trained on biomedical datasets^17,33,34^, EchoCLIP is a model specific for echocardiography, the most common modality for cardiovascular imaging and not represented in prior foundation model training. While echocardiography still needs to be interpreted by expert cardiologists, given the rapid expansion of availability of ultrasound technology and the development of complementary technologies to allow novices to perform cardiac ultrasound^35^, models such as EchoCLIP have the potential to improve access to cardiac imaging and image interpretation. One of the most time-consuming and challenging assessments is distinguishing between natural variation versus change in the disease state that might warrant changes in the treatment plan. Such evaluations often require meticulously comparing current and historical imaging side by side and can be highly variable across different cardiologists. By using EchoCLIP to directly compare studies, clinicians can derive a quantitative visual assessment of differences. Such an automated AI assessment can alert clinicians’ attention toward specific studies to more carefully evaluate clinical changes.

While specialist models still perform better on specific, narrowly defined tasks^29^, the performance of EchoCLIP on external validation data confirms its ability to assess cardiac function with accuracy similar to blinded human performance as well as many previously developed supervised learning models^6,7,36–38^. EchoCLIP achieves an MAE of 7.1% on external validation of LVEF prediction, while previous video-based LVEF AI models achieve an MAE of 6.0% and image-based AI models achieve an MAE of just 9.9%^28^. Differences in EchoCLIP’s performance on internal and external test datasets are likely due to differences in the LVEF evaluation technique across institutions (Supplementary Fig. 4). While statistically significant in large datasets, the error in model predictions across LVEF values or measurement approaches is less than clinical variability^29^, suggesting that different training data with a different LVEF measurement approach would have a modest differential effect. The distribution of LVEF values from model inference is continuous without preference for certain measurements, suggesting that human biases are smoothed out in the model embedding space (Supplementary Fig. 5).

Important limitations of this work include the use of an image encoder instead of a video encoder when echocardiography videos contain important motion-based information and the use of only the apical-four-chamber view, which, although is the most common and informative standard view, does not capture information with regard to Doppler velocities and structures only present in other views. In this work, as well as previous work^8,9^, it is clear that there are image-based features that can be a partial surrogate for information not directly interrogatable without video or from different views. For example, sphericity and dilation of the left ventricle can be identifiable from images alone and suggest decreased cardiac function although true assessment of LVEF requires video information. Valve calcification can hint at stenosis or coronary artery disease^8,9,39^ that is not directly present in the image. Future work will incorporate video encoders and different measurement techniques and will leverage multiple views from the same echocardiographic study to provide more holistic AI models for heart health. Enhancements such as upgrading EchoCLIP’s visual encoder from an image-based model to a video-based model, adapting EchoCLIP for visual question answering, and implementation of automatic report generation are potential directions for future research. Finally, important open questions remain in the testing of foundation models before regulatory approval and eventual clinical use.

Our results encourage further exploration of vision–language foundation models for cardiology and medicine generally. Clinical databases provide large bodies of information about health, while different imaging modalities provide adjunctive ancillary information that might improve our understanding of cardiovascular health. Further efforts remain to leverage larger datasets and more versatile model architectures to better capture and distill medical information.

## Methods

### Data curation

The Cedars-Sinai Medical Center echocardiography laboratory performs clinical echocardiography for a wide range of indications, ranging from asymptomatic preoperative screening to evaluation for open heart surgery or heart transplant. Over the course of a standard, full, resting echocardiogram study, 50–150 videos and images are acquired that visualize the heart from different angles, locations and with different imaging modes (two-dimensional images, tissue Doppler images and color Doppler images). Each echocardiogram study corresponds to a unique patient and a unique visit, but multiple similar videos may be obtained from each view acquired during the study. For EchoCLIP, we focused on the apical-four-chamber view (one of the most common and well-acquired ultrasound views) and organized a dataset of 1,032,975 unique video–caption pairs from 224,685 echocardiogram studies across 99,870 patients, collected between 2011 and 2022. Our laboratory developed high-throughput tools to query echocardiogram videos and their metadata from Cedars-Sinai’s internal databases at scale, view and classify videos and link them to associated structured reporting from cardiologists. DICOM images were queried from a Hyland vendor-neutral archive, linked to interpretations created by trained cardiologists using Syngo Dynamics and converted to AVI video files using PyDICOM before model training and inference.

Data were split by patient into training, validation and internal test datasets. The training data contained 921,981 videos from 84,990 patients, the validation set contained 10,000 videos from 5,358 patients and the internal test set contained 100,994 videos from 10,001 patients. A random subset (*n* = 5,000) of the publicly released EchoNet-Dynamic dataset from Stanford Healthcare was used as an external test set. An automated preprocessing workflow was undertaken to remove extraneous text, ECG and respirometer information and other information outside of the scanning sector. The input data were represented as standardized 224 × 224-pixel RGB videos for model training. This research was approved by the Cedars-Sinai Medical Center (study no. 00001409) and Stanford Healthcare Institutional Review Boards (study no. 43721). A waiver of consent was obtained for the use of retrospective de-identified data.

### Model design and training

Model design and training was conducted in Python using the PyTorch deep-learning library. Our training code is a fork of the OpenCLIP repository^28^. To find the best training configuration, we evaluated a variety of model architectures and training procedures. We tested training with random initialization, initializing the model with CLIP weights, using a convolutional architecture for the image encoder, using a vision transformer for the image encoder, applying random patch dropout to image inputs and using three different text tokenization methods (Supplementary Table 5), with the final EchoCLIP model use the ConvNeXt architecture^26^ for the image encoder and a decoder-only transformer for the text encoder. We initialize our model with weights pretrained on LAION-400M. We trained for 50 epochs, minimizing the original CLIP loss. The CLIP loss incentivizes the video and text encoders to make the embeddings of paired videos and reports as similar as possible, while making the embeddings of unpaired videos and reports as different as possible (Fig. 1a). This training objective is, notably, all that is required to make the two models learn to encode their inputs into semantically meaningful vector embeddings.

We warmed up to an initial learning rate of 5 × 10^−5^ over the course of the first 2,000 training steps and then cosine decayed to zero over the course of the training run. We used a batch size of 1,024 and trained on two Nvidia RTX A6000 48 GB GPUs for approximately 2 weeks. During training, a random frame was extracted from each video and passed to the image encoder. A random frame from each video was used for each epoch as a form of data augmentation. Model checkpoints were saved after every epoch. At the end of training, the model checkpoint with the lowest mean cross-modal retrieval rank on the validation set was selected for testing. Before computing the cosine similarity between vector embeddings, we always divide them by their norms to ensure that they have the same magnitude. This means that the cosine similarity metric always returns a value between −1 and 1.

### Text tokenization

A number of text tokenization schemes were tested (Supplementary Table 1). EchoCLIP was trained using text tokenized by a BPE tokenizer^27^ pretrained on the GPT2 data corpus, which encoded echocardiography reports with a mean of 530.3 (±154.7) tokens per report. Due to the context length limit of 77 tokens imposed by fine-tuning from CLIP weights, EchoCLIP was trained on snippets of reports rather than their full text. For EchoCLIP-R, we noted that the echocardiography report text is often highly structured and repetitive, as they are typically generated in a ‘fill-in-the-blank’ fashion according to a predetermined template given to the cardiologist at the time of interpretation. The templated nature of the reports means that a small number of unique phrases and sentences appear very frequently in the final report text with only slight variations. A custom-built tokenizer was designed to take advantage of this observation and allowed us to aggressively compress the report text. This meant that whole reports could be inputted when training EchoCLIP-R, improving its retrieval capabilities compared to EchoCLIP at the cost of slight degradation in classification and estimation capabilities.

Instead of searching for exact vocabulary matches in the report text, our custom-built template tokenizer uses regular expressions to allow nearly similar lines of text to be efficiently encoded. For example, the text ‘Moderate left ventricular hypertrophy. Left ventricular ejection fraction is 60%’ is converted into tokens indicating either cardiac structure or function (such as ‘<_ left ventricular hypertrophy>’, ‘<left ventricular ejection fraction is _%>’) as well as indicating severity (‘mild’, ‘moderate’ or ‘severe’) or quantity (60%, 2.5 cm, 40 cm s^−1^). By doing this, we were able to capture most of the variance present in our text reports with a vocabulary containing only 770 words and phrases, in addition to extra tokens for handling numbers and severity terms. After applying this custom tokenizer, the mean length of a tokenized report was brought down to just 63.8 (±26.7) tokens, an approximate ninefold reduction compared to using CLIP’s original BPE tokenizer. We additionally tested a model that used a BPE tokenizer pretrained on echocardiography reports but found that it failed to outperform the model trained using our custom solution.

Using EchoCLIP-R embeddings, we can perform a search within our test set to find images or reports that are semantically similar to a given query image or report. To do this, we simply sort the embeddings of all candidate images or reports by their cosine similarity to the embedding of a query image or report. The embedding space was normalized to unit vectors before calculation of cosine similarity to be insensitive to projection magnitude. If the model and dataset were theoretically perfect, we would expect the image or report that is officially paired with the query image or report to be ranked first in the list. We report the mean rank number as a metric of accuracy. This allows us to characterize EchoCLIP-R’s retrieval abilities in two settings: image-to-report and report-to-image. We choose a single random video from each study to represent the whole study in these ranking tests to simplify the implementation. To obtain a single value that represents a model’s overall retrieval ability, we define the MCMRR as the average of both the mean image-to-report retrieval rank and the mean report-to-image retrieval rank. MCMRR values for both EchoCLIP and EchoCLIP-R are shown in Table 2.

To evaluate the model’s ability to identify unique patients, we computed the similarity between many random pairs of EchoCLIP-R’s image embeddings and then treated those similarity values as if they were continuous probability predictions meant to classify whether both images in the pair came from the same patient. To visualize patient trajectories before or after heart transplantation or cardiac surgery, we first collected all the echocardiogram images within 200 days before or after the procedure date. These images were grouped by study and then embeddings were produced for each video using EchoCLIP-R. The earliest study within the 200-day window was taken as a baseline and then each following study within the window was assigned a similarity score computed by taking the average similarity between all possible pairs of videos from the baseline study and the study in question. This was repeated for all patients who had undergone heart transplantation or cardiac surgery in our test set who also had at least one echocardiography study performed before and after the date of the procedure. These study-level ‘similarity timelines’ were then plotted together, resampled and averaged to create Fig. 3c,d. These study-level similarity scores can also treated as continuous probability predictions for whether a given study was acquired before or after the procedure date, allowing us to calculate an AUC score that quantifies EchoCLIP-R’s ability to detect the effects of such procedures. Multiple surgical characteristics and approaches were analyzed by subset analysis with similar results (Supplementary Figs. 6 and 7).

### Adapting EchoCLIP to classification and regression tasks

Despite only training to encode images and report text as semantically meaningful vector embeddings, EchoCLIP was adapted to perform both classification and regression tasks. For each classification task, we followed the approach of the original CLIP paper and constructed text prompts describing a positive case. Then, we obtained an embedding of those prompts using EchoCLIP’s text encoder and computed the cosine similarity between them and the embeddings of the videos in our test set. In the case of multiple semantically equivalent prompts being used for a binary classification task, we average the similarity across all prompts and then averaged again over the first 20 frames of the video (at temporal stride 2). We treat this final average similarity score as a continuous probability prediction. Hyperparameters of number of frames sampled per video, stride (frame count between sampled frames) and number of averaged embeddings were evaluated to optimize model performance (Supplementary Tables 4 and 5).

**Extended Data Fig. 1:**
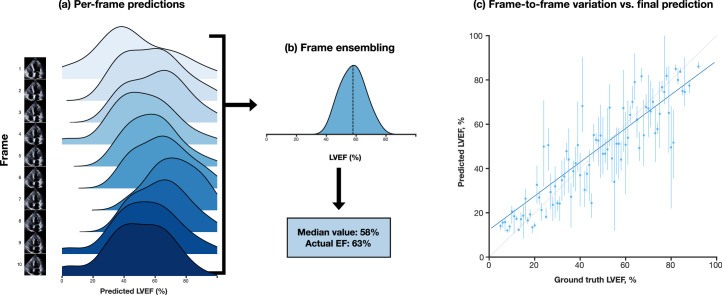
Frame level ensembling. (**a**) Distribution of EchoCLIP left ventricular ejection fraction (LVEF) from individual frames of an echocardiogram video, which are averaged to (**b**) a video-level distribution of LVEF prediction. (**c**) Scatter-plot of subset of test dataset (n = 1,000 predictions from 100 videos and 10 frames per video) representing predicted vs. ground-truth LVEF. Each point represents the final predicted values and whiskers represent the range of frame level predictions for that video.

For regression tasks, we generated a collection of variations on a base text prompt by only changing the relevant value in the text (Supplementary Fig. 8). For instance, variants of the prompt ‘The left ventricular ejection fraction is estimated to be X%’ or ‘LV ejection fraction is X%’ were generated for all integer values between 0 and 100. These variations on the base prompt are then embedded using EchoCLIP’s text encoder. The cosine similarity between these prompt embeddings and the embeddings of each of the first 20 frames of all test-set videos (extracted with temporal stride of 2) is computed. The candidate values are then ranked for each frame according to their corresponding prompt embeddings’ similarity to the frame embeddings and the bottom 80% of the values are discarded. The remaining 20% of the values are averaged along the frames dimension, leaving 20 potential prediction values ordered from most likely (on average across all frames) to least likely. We found, empirically, that taking the median of these 20 values results in the most accurate predictions. This process is illustrated in Extended Data Fig. 1.

For EchoCLIP, a systematic search through relevant phrases present in the echocardiography report template file was conducted to manually construct the base prompts for each task. For EchoCLIP-R, we noted that using this approach resulted in severely degraded performance. We believe this to be the result of short, single-phrase prompts being out-of-distribution for EchoCLIP-R as it was trained exclusively using full-length reports. To address this, we tested an alternate prompting strategy for EchoCLIP-R, where the base prompts are entire reports sampled from videos in the validation set that have the desired labels. As an example of how this works for a regression task, the base LVEF estimation prompts for EchoCLIP-R were chosen by randomly sampling up to ten reports from the validation set for each ground-truth LVEF value between 1 and 100. This way, EchoCLIP-R has in-distribution ‘example reports’ from the validation set to compare the query images against, instead of being forced to encode much shorter prompts that are nothing like what it saw during training. For binary tasks, 200 reports containing a positive label for the task are sampled from the validation set and used as base prompts. We found that this ‘sampled prompts’ strategy substantially improved EchoCLIP-R’s performance on classification and regression tasks (Table 2).

All text prompts used for the evaluation of EchoCLIP are published in the project’s code repository, a link to which is included in Supplementary Fig. 8. Ground-truth labels are extracted from the clinical reports and used to calculate AUC and other performance metrics.

### Interpretation techniques

Code for saliency mapping with PromptCAM was written in Python with dependencies on PyTorch and NumPy packages. Modifying the optimization function of the integrated gradients method, PromptCAM maximizes the cosine similarity as the objective function between image-based regions of interest with the text prompt. Prompts describing common cardiac structures were used to test whether EchoCLIP ‘pays attention’ to relevant cardiac structures in echocardiogram images. UMAP was applied using the umap-learn Python package. EchoCLIP image embeddings for each video in the test set were processed to demonstrate how clusters associated with different cardiovascular diseases, disease states and measurements are present. The n_neighbors parameter was set to the maximum allowed value of 200 and the min_distance parameter was set to the maximum allowed value of 1.0.

### Reporting summary

Further information on research design is available in the Nature Portfolio Reporting Summary linked to this article.

## Online content

Any methods, additional references, Nature Portfolio reporting summaries, source data, extended data, supplementary information, acknowledgements, peer review information; details of author contributions and competing interests; and statements of data and code availability are available at 10.1038/s41591-024-02959-y.

### Supplementary information

Supplementary Information Supplementary Tables 1–5 and Figs. 1–8.

Reporting Summary

Supplementary Video Representative video of EchoCLIP inference in-text retrieval across an echocardiogram study.

## Figures and Tables

**Extended Data Table 1:**
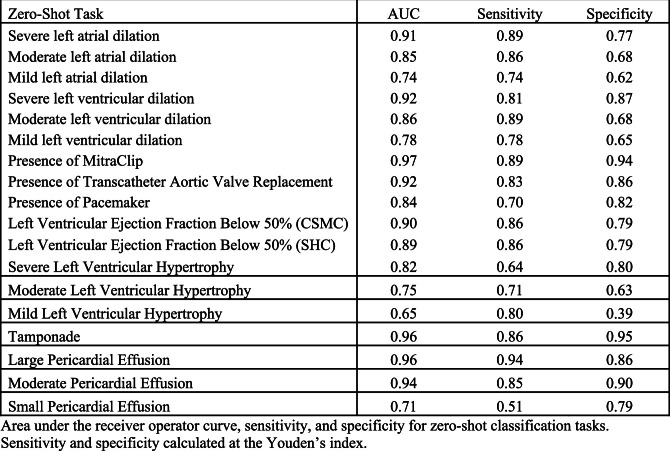
AUC, sensitivity, and specificity for EchoCLIP zero-shot prediction tasks

Area under the receiver operator curve, sensitivity, and specificity for zero-shot classification tasks. Sensitivity and specificity calculated at the Youden’s index.

## Data Availability

The dataset of videos and reports used to train EchoCLIP is not publicly available due to its potentially identifiable nature; however, EchoNet-Dynamic, the dataset that we used for external validation, is publicly available at https://echonet.github.io/dynamic/.

## Code availability

Our model weights, evaluation prompts, and demonstration code are available on GitHub at https://github.com/echonet/echo_CLIP.
