# Supplementary material for: Vision–language foundation model for echocardiogram interpretation
Source: Nat Med. 2024 Apr 30;30(5):1481–8. doi: 10.1038/s41591-024-02959-y (PMC11108770; doi:10.1038/s41591-024-02959-y)
Supplement: Supplementary file 1 — Supplementary Tables 1–5 and Figs. 1–8. [file 41591_2024_2959_MOESM1_ESM.pdf]

nature medicine

Article

https://doi.org/10.1038/s41591-024-02959-y

Vision–language foundation model for echocardiogram interpretation

In the format provided by the authors and unedited

SUPPLEMENTARY MATERIALS

Table of Contents

SUPPLEMENTARY TABLE 1: BENCHMARK COMPARISONS WITH OTHER MULTI-MODAL MODELS AND TRAINING PARAMETERS 2

SUPPLEMENTARY TABLE 2: PERFORMANCE IN AGE AND SEX STRATIFIED POPULATIONS 3

SUPPLEMENTARY TABLE 3: PERFORMANCE METRICS FOR LVEF AT DIFFERENT THRESHOLDS 4

SUPPLEMENTARY TABLE 4: HYPERPARAMETER SWEEP OF FRAME COUNT AND STRIDE 5

SUPPLEMENTARY TABLE 5: HYPERPARAMETER SWEEP OF NUMBER OF FRAMES TO ENSEMBLE 6

SUPPLEMENTARY FIGURE 1: BLAND-ALTMAN PLOTS FOR ZERO-SHOT REGRESSION TASKS 7

SUPPLEMENTARY FIGURE 2: RELATIONSHIP OF DIFFERENCE IN LEFT VENTRICULAR EJECTION FRACTION BETWEEN STUDIES AND COSINE SIMILARITY BETWEEN STUDIES 8

SUPPLEMENTARY FIGURE 3: UNIFORM MANIFOLD APPROXIMATION AND PROJECTION (UMAP) OF THE SECOND TO LAST LAYER OF THE IMAGE ENCODER OF ECHOCLIP 9

SUPPLEMENTARY FIGURE 4: DISTRIBUTION OF LEFT VENTRICULAR EJECTION FRACTION BETWEEN STUDIES ACROSS TWO SITES 10

SUPPLEMENTARY FIGURE 5: DISTRIBUTION OF ECHOCLIP INFERENCE OF LEFT VENTRICULAR EJECTION FRACTION 11

SUPPLEMENTARY FIGURE 6: QUANTITATIVE CHANGE IN ECHOCLIP EMBEDDING ACROSS TIME BEFORE/AFTER TRANSPLANT AND SURGERY (ONE VIDEO PER STUDY) 12

SUPPLEMENTARY FIGURE 7: PATIENT TRAJECTORIES FOR VARIOUS CARDIAC SURGERIES BASED ON ECHOCLIP EMBEDDINGS 13

SUPPLEMENTARY FIGURE 8: TEXT PROMPTS 14

Supplementary Table 1: Benchmark comparisons with other multi-modal models and training parameters

	Image encoder	Tokenization	Mean text-to-image rank	Mean image-to-text rank	Text-to-image retrieval, R@10	Image-to-text retrieval, R@10	MCMRR
CLIP	ViT-B-32	CLIP BPE	10,743.70	10,742.20	0.001	0	10,743.00
EchoCLIP	ConvNeXt-Base	CLIP BPE	560.4	582.1	0.16	0.166	571.3
EchoCLIP-R	ConvNeXt-Base	Template tokenizer	**203.1**	**209.1**	**0.343**	**0.333**	**206.1**
ViT backbone	ViT-B-32	CLIP BPE	619.3	621.6	0.147	0.15	620.5
Random initialization	ViT-B-32	CLIP BPE	867.4	863.7	0.106	0.112	865.6
With patch dropout	ViT-B-32	CLIP BPE	829.0	833.5	0.11	0.118	831.2
EchoBPE	ConvNeXt-Base	EchoBPE1K	291.4	260.2	0.288	0.288	275.8

	Ejection fraction, MAE	Heart failure, AUC	Pacemaker, AUC	Impella, AUC	TAVR, AUC	MitraClip, AUC
CLIP	20.76 (20.69-20.83)	0.49 (0.49-0.50)	0.51 (0.51-0.52)	0.63 (0.60-0.67)	0.46 (0.46-0.47)	0.53 (0.52-0.54)
EchoCLIP	**8.35 (8.30-8.40)**	**0.90 (0.89-0.90)**	**0.84 (0.84-0.84)**	0.98 (0.98-0.99)	**0.92 (0.91-0.92)**	**0.97 (0.97-0.97)**
EchoCLIP-R	16.89 (16.83-16.95)	0.50 (0.50-0.51)	0.66 (0.65-0.66)	0.42 (0.38-0.46)	0.52 (0.51-0.52)	0.81 (0.81-0.82)
ViT backbone	9.61 (9.56-9.66)	0.87 (0.87-0.87)	**0.84 (0.84-0.85)**	**0.99 (0.98-0.99)**	0.91 (0.91-0.92)	**0.97 (0.97-0.97)**
Random initialization	11.83 (11.77-11.90)	0.70 (0.69-0.70)	0.69 (0.68-0.69)	0.97 (0.96-0.97)	0.83 (0.83-0.84)	0.94 (0.94-0.95)
With patch dropout	11.65 (11.58-11.71)	0.77 (0.77-0.77)	0.73 (0.72-0.73)	0.91 (0.89-0.93)	0.80 (0.80-0.81)	0.89 (0.89-0.90)
EchoBPE	10.28 (10.23-10.35)	0.76 (0.76-0.76)	0.77 (0.77-0.78)	**0.99 (0.98-0.99)**	0.86 (0.86-0.87)	0.96 (0.96-0.96)

	Significantly elevated RAP, AUC	Severe LV dilation, AUC	Severe RV dilation, AUC	Severe LA dilation, AUC	Severe RA dilation, AUC	PA pressure, MAE	Severely elevated PA pressure, AUC
CLIP	0.53 (0.53-0.54)	0.58 (0.57-0.59)	0.56 (0.55-0.57)	0.54 (0.53-0.55)	0.59 (0.57-0.61)	16.84 (16.77-16.91)	0.52 (0.51-0.52)
EchoCLIP	**0.83 (0.82-0.83)**	**0.92 (0.92-0.93)**	**0.92 (0.91-0.92)**	0.91 (0.90-0.92)	**0.97 (0.97-0.98)**	**10.81 (10.75-10.88)**	**0.85 (0.84-0.85)**
EchoCLIP-R	0.48 (0.48-0.49)	0.87 (0.86-0.87)	0.81 (0.80-0.82)	0.83 (0.82-0.84)	0.81 (0.80-0.82)	17.45 (17.38-17.52)	0.61 (0.60-0.61)
ViT backbone	0.82 (0.81-0.82)	0.90 (0.90-0.91)	0.91 (0.90-0.91)	**0.93 (0.92-0.93)**	0.96 (0.96-0.97)	11.26 (11.19-11.34)	0.81 (0.81-0.81)
Random initialization	0.66 (0.65-0.66)	0.90 (0.89-0.91)	0.82 (0.81-0.83)	0.87 (0.86-0.87)	0.90 (0.89-0.91)	15.66 (15.58-15.73)	0.63 (0.62-0.63)
With patch dropout	0.63 (0.62-0.64)	0.86 (0.86-0.87)	0.77 (0.76-0.78)	0.85 (0.84-0.86)	0.89 (0.88-0.90)	19.81 (19.73-19.89)	0.59 (0.58-0.59)
EchoBPE	0.69 (0.69-0.70)	0.91 (0.90-0.91)	0.84 (0.84-0.85)	0.86 (0.85-0.86)	0.93 (0.92-0.94)	22.66 (22.58-22.74)	0.80 (0.80-0.81)

Supplementary Table 2: Performance in Age and Sex Stratified Populations

Cohort	LVEF, MAE	PAP, MAE	TAVR, AUC	Mitraclip, AUC	Pacemaker, AUC
**Male**	8.47 (8.40-8.53)	10.96 (10.87-11.06)	0.92 (0.92-0.93)	0.97 (0.97-0.97)	0.84 (0.84-0.85)
**Female**	8.09 (8.02-8.16)	10.59 (10.49-10.68)	0.91 (0.91-0.92)	0.98 (0.97-0.98)	0.84 (0.83-0.85)
**Less than 45 years old**	8.15 (8.02-8.29)	9.23 (9.06-9.41)	0.94 (0.89-0.98)	0.97 (0.96-0.98)	0.85 (0.84-0.87)
**45-60 years old**	7.82 (7.71-7.91)	9.82 (9.65-9.98)	0.87 (0.84-0.89)	0.97 (0.96-0.98)	0.87 (0.86-0.88)
**60-75 years old**	8.09 (8.01-8.18)	10.87 (10.76-10.99)	0.92 (0.91-0.92)	0.96 (0.96-0.97)	0.81 (0.81-0.82)
**Greater than 75 years old**	8.86 (8.78-8.95)	11.75 (11.63-11.86)	0.87 (0.86-0.87)	0.97 (0.97-0.98)	0.83 (0.82-0.83)
**Atrial fibrillation**	9.06 (8.87-9.24)	13.90 (13.64-14.16)	0.89 (0.88-0.90)	0.96 (0.94-0.97)	0.80 (0.79-0.82)

Supplementary Table 3: Performance metrics for LVEF at different thresholds

Zero-Shot Task	AUC	Sensitivity	Specificity
Left Ventricular Ejection Fraction Below 50% (CSMC)	0.90 (0.90 - 0.90)	0.86 (0.86 - 0.86)	0.79 (0.79 - 0.80)
Left Ventricular Ejection Fraction Below 50% (SHC)	0.89 (0.88 - 0.90)	0.86 (0.86 - 0.86)	0.79 (0.79 - 0.80)
Left Ventricular Ejection Fraction Below 40% (CSMC)	0.93 (0.93 - 0.93)	0.95 (0.95 - 0.95)	0.74 (0.73 - 0.74)
Left Ventricular Ejection Fraction Below 40% (SHC)	0.94 (0.93 - 0.95)	0.95 (0.95 - 0.95)	0.74 (0.73 - 0.74)
Left Ventricular Ejection Fraction Below 30% (CSMC)	0.96 (0.96 - 0.96)	0.98 (0.98 - 0.98)	0.65 (0.64 - 0.66)
Left Ventricular Ejection Fraction Below 30% (SHC)	0.96 (0.95 - 0.97)	0.98 (0.98 - 0.98)	0.65 (0.64 - 0.66)

Supplementary Table 4: Hyperparameter sweep of frame count and stride

Effect of frame count and temporal stride on validation EF MAE					
	**Stride=1**	**Stride=2**	**Stride=3**	**Stride=4**	**Stride=5**
**1 frames**	10.16	10.16	10.16	10.16	10.16
**5 frames**	9.66	9.54	9.63	9.42	10.16
**10 frames**	8.86	8.92	8.78	8.86	9.24
**20 frames**	8.5	**8.49**	8.53	8.53	8.57
**40 frames**	8.55	8.54	8.54	8.57	8.57

Supplementary Table 4: Hyperparameter sweep of number of sampled frames and sampling rate between frames (stride) as evaluated on the validation split by mean absolute error (MAE) in compared to ground truth left ventricular ejection fraction (LVEF)

Supplementary Table 5: Hyperparameter sweep of number of frames to ensemble

Ensembling Approach	LVEF MAE	PA Pressure MAE
Top 1 Embedding	**11.18**	**14.68**
Top 10% of Embeddings	**8.57**	**11.12**
Top 20% of Embeddings	**8.36**	**10.84**

Supplementary Figure 1: Bland-Altman Plots for Zero-Shot Regression Tasks

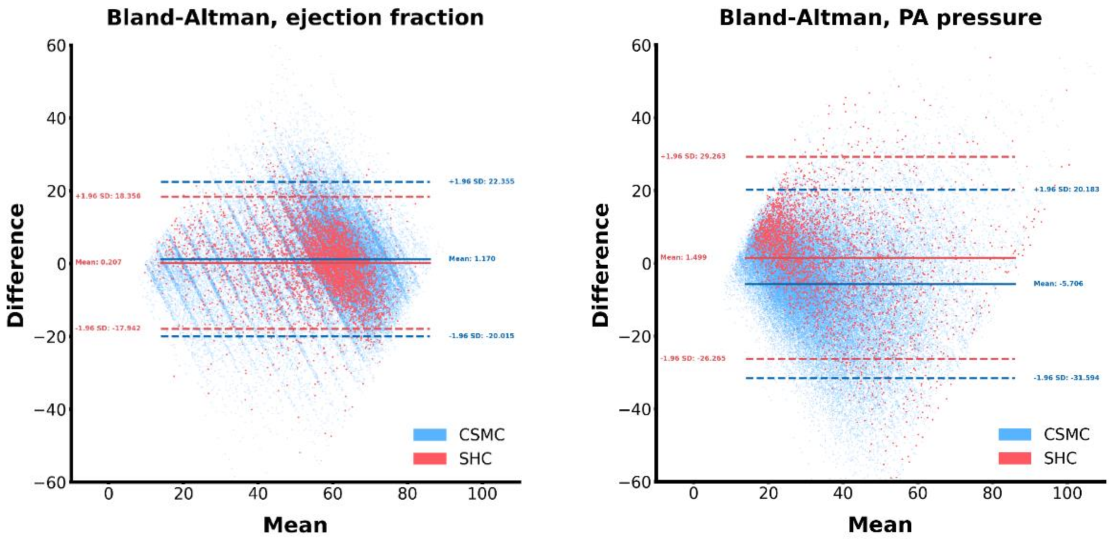

Bland-Altman plot for test datasets from Cedars-Sinai Medical Center (blue, n = 100,994) and Stanford Healthcare (red, n = 5,000). X axis is the mean of predicted and ground truth and y axis is the difference between predicted and ground truth. Left ventricular ejection fraction as a percentage (%) and pulmonary artery (PA) pressure in units of mmHg.

Supplementary Figure 2: Relationship of Difference in Left Ventricular Ejection Fraction Between Studies and Cosine Similarity Between Studies

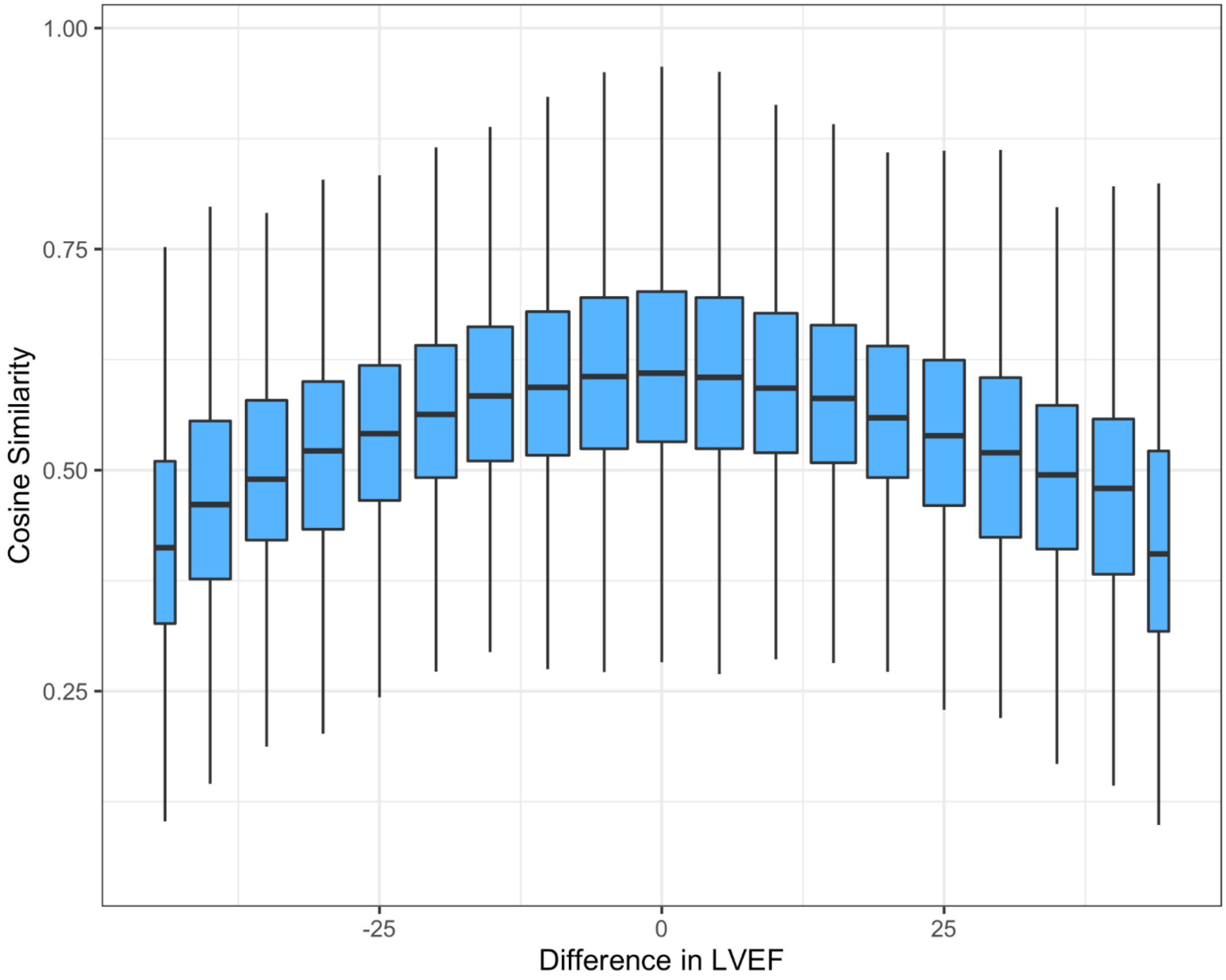

Boxplot of cosine similarity across range of difference between predicted and clinician-assessed left ventricular ejection fraction (LVEF). Boxplot shows the 25th percentile to 75th percentile in the box, with the median shown in the middle. The whispers show the range from maximum to minimum cosine similarity up to 1.5*IQR outside the boxplot.

Supplementary Figure 3: Uniform manifold approximation and projection (UMAP) of the second to last layer of the image encoder of EchoCLIP

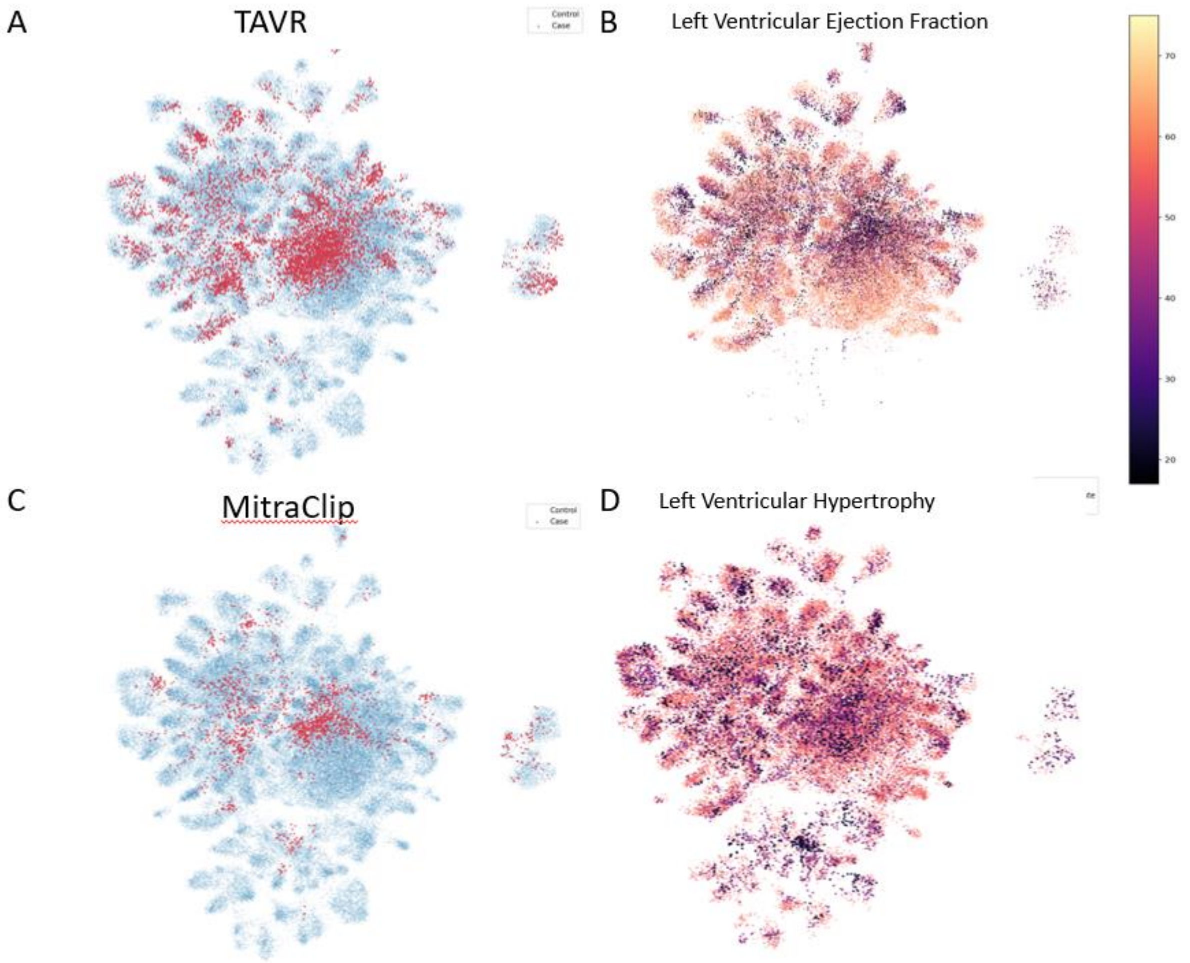

Supplementary Figure 4: Distribution of Left Ventricular Ejection Fraction Between Studies Across Two Sites

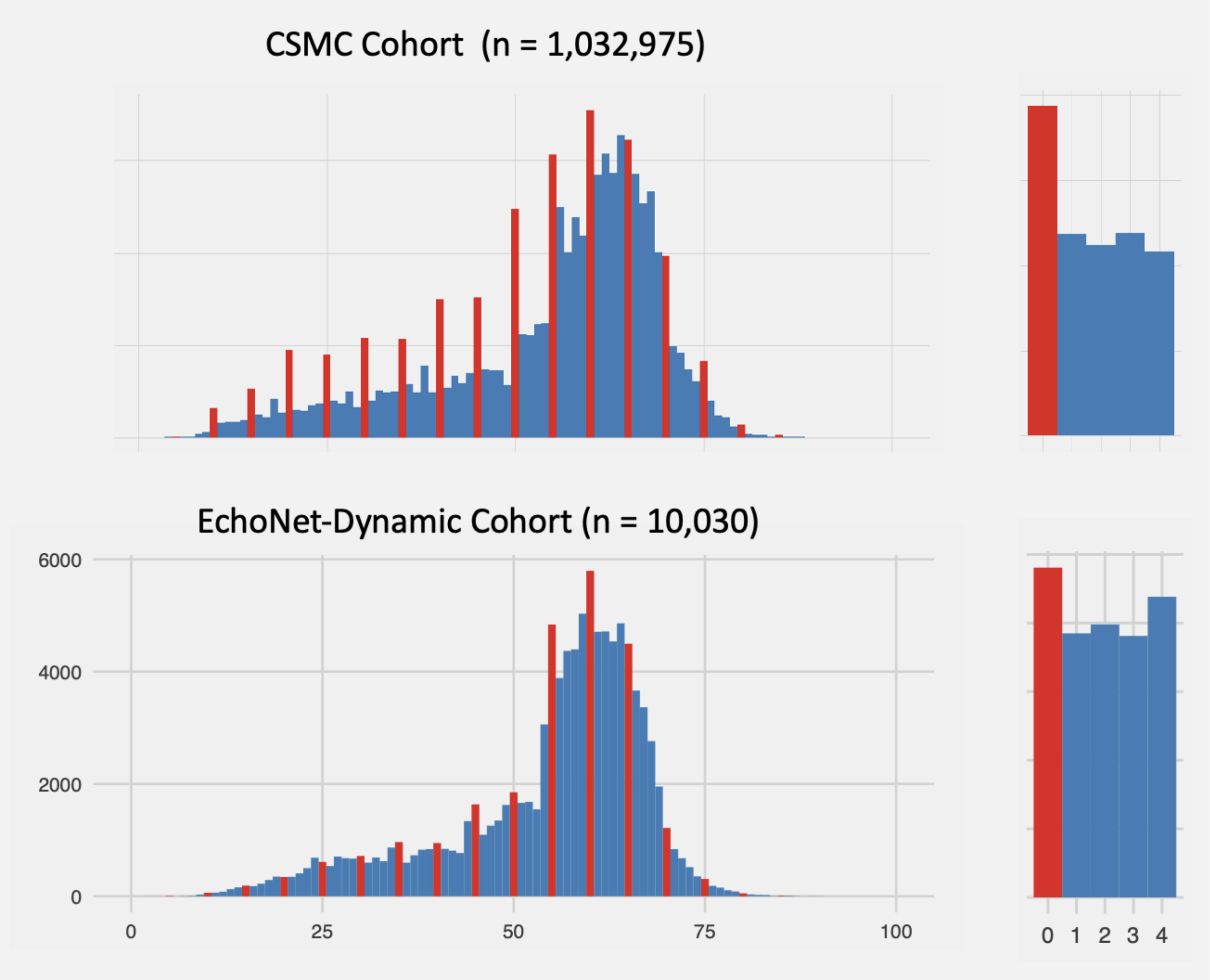

When using the full echocardiography database's data, a variety of methods of left ventricular ejection fraction (LVEF) are possible. There is a higher proportion of reported LVEF ending in 0% or 5% (35%,40%,45%, etc) suggesting a component of visual estimation in addition to formula driven calculations. This different is not as commonly seen in the EchoNet-Dynamic cohort, which is a subset of echocardiogram studies from Stanford Healthcare and chosen with having Simpson's method of disks calculations.

Supplementary Figure 5: Distribution of EchoCLIP Inference of Left Ventricular Ejection Fraction

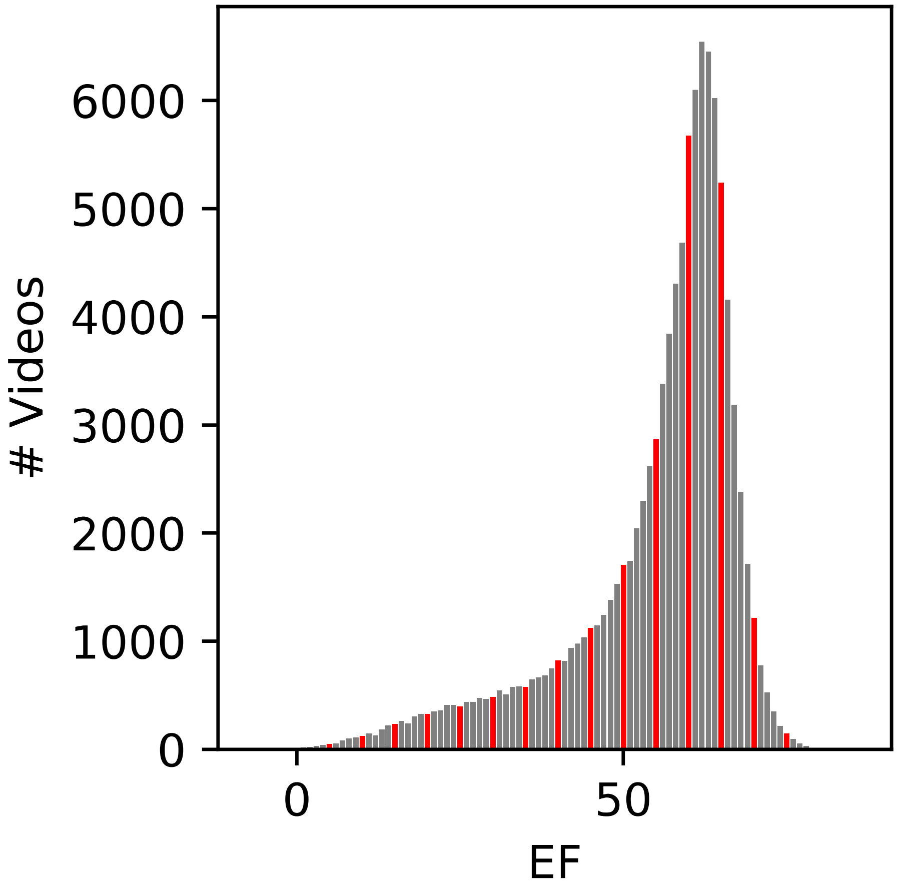

Supplementary Figure 6: Quantitative Change in EchoCLIP Embedding Across Time Before/After Transplant and Surgery (One Video Per Study)

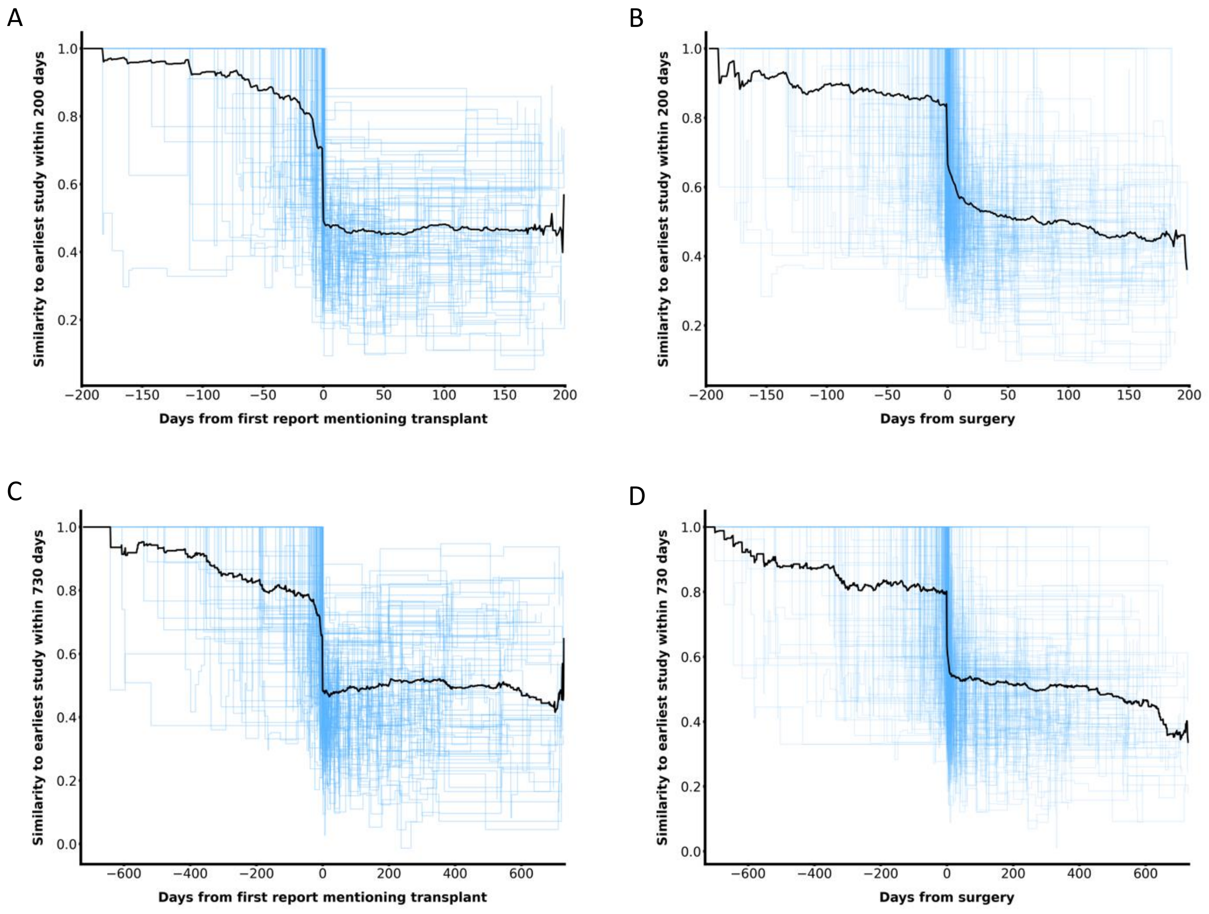

Plotting the similarity of patients over time with a single random apical-4-chamber per study. All studies were compared to the first from the same patient over a window of 400 days. Patients either had a heart transplant (A) or other major cardiac surgery (B), and the window is centered on the date of these events. (C,D). Extended horizon visualization at two years (730 days) before and after the critical event.

Supplementary Figure 7: Patient trajectories for various cardiac surgeries based on EchoCLIP embeddings

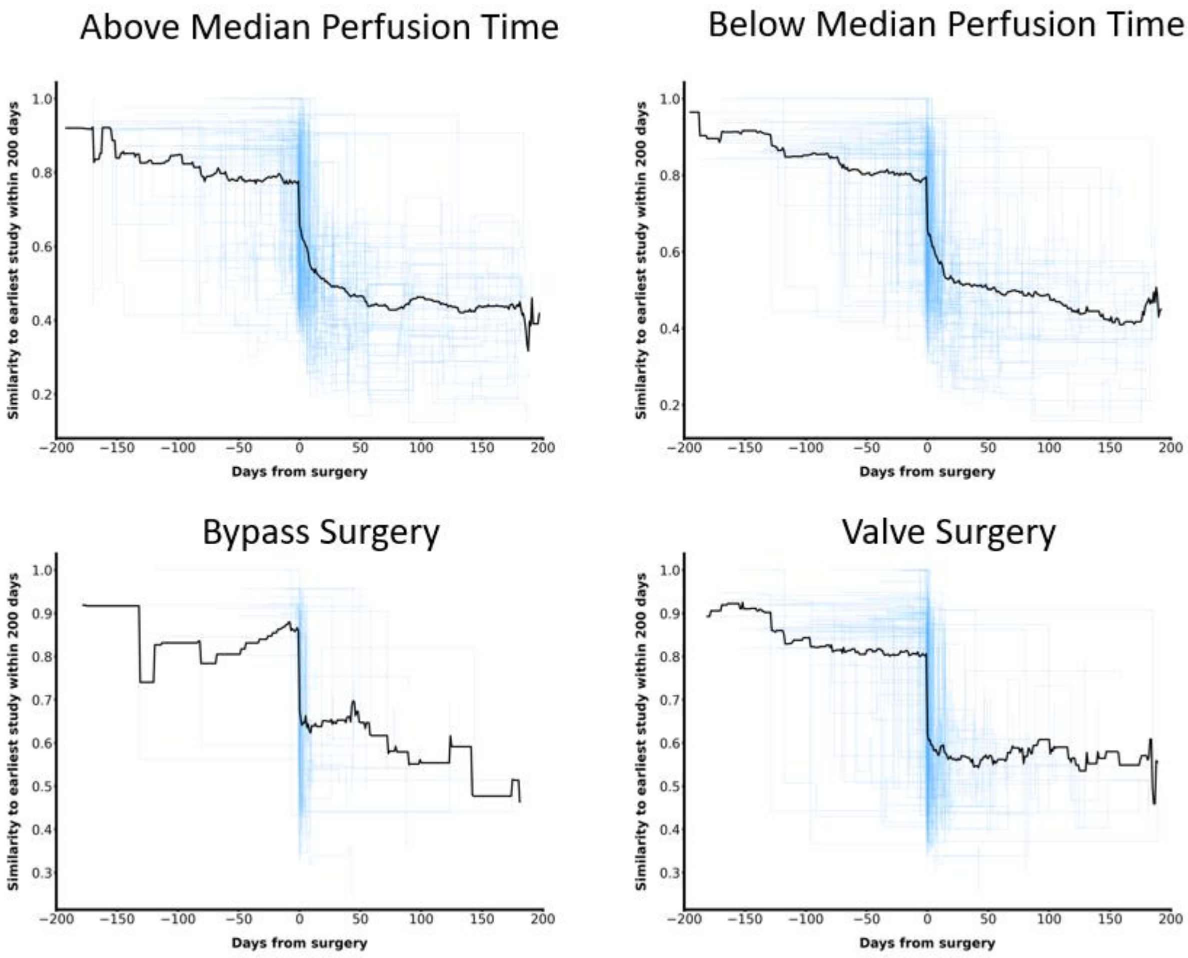

Supplementary Figure 8: Text Prompts

prompts_used.json


```json
{
    "ejection_fraction": {
        "mode": "regression",
        "label_sources": [
            "THE LEFT VENTRICULAR EJECTION FRACTION IS ESTIMATED TO BE <#>% ",
            "LV EJECTION FRACTION IS <#>%. ",
        ],
        "range": [0, 100],
    },
    "pacemaker": {
        "mode": "binary",
        "label_sources": [
            "ECHO DENSITY IN RIGHT VENTRICLE SUGGESTIVE OF CATHETER, PACER LEAD, OR ICD LEAD. ",
            "ECHO DENSITY IN RIGHT ATRIUM SUGGESTIVE OF CATHETER, PACER LEAD, OR ICD LEAD. ",
        ],
    },
    "impella": {
        "mode": "binary",
        "label_sources": [
            "AN IMPELLA CATHETER IS SEEN AND THE INLET AREA IS <#>CM FROM THE AORTIC VALVE AND DOES NOT INTERFERE WITH NEIGHBORING STRUCTURES, CONSISTENT WITH CORRECT IMPELLA POSITIONING. THERE IS DENSE TURBULENT COLOR FLOW ABOVE THE AORTIC VALVE, CONSISTENT WITH CORRECT OUTFLOW AREA POSITION ",
            "AN IMPELLA CATHETER IS SEEN ACROSS THE AORTIC VALVE AND IS TOO CLOSE TO OR ENTANGLED IN THE PAPILLARY MUSCLE AND SUBANNULAR STRUCTURES SURROUNDING THE MITRAL VALVE; REPOSITIONING RECOMMENDED. ",
            "AN IMPELLA CATHETER IS SEEN, HOWEVER THE INLET AREA APPEARS TO BE IN THE AORTA OR NEAR THE AORTIC VALVE; REPOSITIONING IS RECOMMENDED. ",
            "AN IMPELLA CATHETER IS SEEN ACROSS THE AORTIC VALVE AND EXTENDS TOO FAR INTO THE LEFT VENTRICLE; REPOSITIONING RECOMMENDED ",
```


https://github.com/echonet/echo_CLIP/blob/main/prompts_used.json
