# Supplementary material for: Vision–language foundation model for echocardiogram interpretation
Source: Nat Med. 2024 Apr 30;30(5):1481–8. doi: 10.1038/s41591-024-02959-y (PMC11108770; doi:10.1038/s41591-024-02959-y)
Supplement: Supplementary file 2 — Reporting Summary [file 41591_2024_2959_MOESM2_ESM.pdf]

nature portfolio

Corresponding author(s): Christensen et al

Last updated by author(s): 3/26/2024

Reporting Summary

Nature Portfolio wishes to improve the reproducibility of the work that we publish. This form provides structure for consistency and transparency in reporting. For further information on Nature Portfolio policies, see our Editorial Policies and the Editorial Policy Checklist.

Statistics

For all statistical analyses, confirm that the following items are present in the figure legend, table legend, main text, or Methods section.

n/a	Confirmed	
☐	☒	The exact sample size (n) for each experimental group/condition, given as a discrete number and unit of measurement
☐	☒	A statement on whether measurements were taken from distinct samples or whether the same sample was measured repeatedly
☐	☒	The statistical test(s) used AND whether they are one- or two-sided<br>*Only common tests should be described solely by name; describe more complex techniques in the Methods section.*
☐	☒	A description of all covariates tested
☐	☒	A description of any assumptions or corrections, such as tests of normality and adjustment for multiple comparisons
☐	☒	A full description of the statistical parameters including central tendency (e.g. means) or other basic estimates (e.g. regression coefficient) AND variation (e.g. standard deviation) or associated estimates of uncertainty (e.g. confidence intervals)
☐	☒	For null hypothesis testing, the test statistic (e.g. F, t, r) with confidence intervals, effect sizes, degrees of freedom and P value noted<br>*Give P values as exact values whenever suitable.*
☒	☐	For Bayesian analysis, information on the choice of priors and Markov chain Monte Carlo settings
☒	☐	For hierarchical and complex designs, identification of the appropriate level for tests and full reporting of outcomes
☒	☐	Estimates of effect sizes (e.g. Cohen's d, Pearson's r), indicating how they were calculated

Our web collection on statistics for biologists contains articles on many of the points above.

Software and code

Policy information about availability of computer code

Data collection	Data was collected from the echocardiography laboratories at Cedars-Sinai Medical Center. Medical images were converted from initial DICOM files into AVI videos files prior to deep learning training. Associated text and patient identifiers were mapped from the electronic healthcare record for training but videos we de-identified prior to input into AI model.
Data analysis	A deep learning algorithm was used to assess the echocardiogram videos. Zero shot tasks were performed with text prompts. Our code and working model is available at https://github.com/echonet/echo-clip. Required software packages for model training and inference include pytorch, torchvision, open_clip_torch, huggingface_hub, tokenizers, opencv-python-headless, pathlib, numpy, re, matplotlib, os, PIL, and scipy.

For manuscripts utilizing custom algorithms or software that are central to the research but not yet described in published literature, software must be made available to editors and reviewers. We strongly encourage code deposition in a community repository (e.g. GitHub). See the Nature Portfolio guidelines for submitting code & software for further information.

Data

Policy information about availability of data

All manuscripts must include a data availability statement. This statement should provide the following information, where applicable:

- Accession codes, unique identifiers, or web links for publicly available datasets
- A description of any restrictions on data availability
- For clinical datasets or third party data, please ensure that the statement adheres to our policy

The dataset of videos and reports used to train EchoCLIP is not publicly available due to its potentially identifiable nature. However, EchoNet-Dynamic, the dataset we used for external validation, is publicly available at https://echonet.github.io/dynamic/.

Research involving human participants, their data, or biological material

Policy information about studies with human participants or human data. See also policy information about sex, gender (identity/presentation), and sexual orientation and race, ethnicity and racism.

Reporting on sex and gender	Cohort demographics including patient sex are described in Table 1. Model performance in sex stratified populations shown in Supplementary Table 2.
Reporting on race, ethnicity, or other socially relevant groupings	The input data for training comes from a large academic medical center with a diverse patient population (demographics shown in Table 1). The race, ethnicity and other socially relevant groupings were not used for model input given recognized biases that might happen if that were an input predictor (Duffy et al. npj Digital Medicine).
Population characteristics	Echocardiograms acquired at Cedars Sinai Medical Center between 2011 and 2022 were used to train the model. Detailed test and training cohort information in Table 1. External validation data from Ouyang et al. Nature 2020 publicly released data and cohort demographics in the prior manuscript.
Recruitment	A waiver of consent was obtained for the use of retrospective de-identified data. Patient data from 2011 to 2022 were used in de-identified format without prospective recruitment.
Ethics oversight	This research was approved by the Cedars-Sinai Medical Center (Study00001409) and Stanford Healthcare Institutional Review Boards (Study 43721). A waiver of consent was obtained for the use of retrospective de-identified data.

Note that full information on the approval of the study protocol must also be provided in the manuscript.

Field-specific reporting

Please select the one below that is the best fit for your research. If you are not sure, read the appropriate sections before making your selection.

☒ Life sciences ☐ Behavioural & social sciences ☐ Ecological, evolutionary & environmental sciences

For a reference copy of the document with all sections, see nature.com/documents/nr-reporting-summary-flat.pdf

Life sciences study design

All studies must disclose on these points even when the disclosure is negative.

Sample size	1,032,975 echocardiograms were collected from 224,685 unique studies performed on 99,870 unique patients. Sample size calculations were not done prior to the development of the model as we sought to optimize for the largest possible training dataset size. The sample size was chosen based off of availability of echocardiogram videos for training at the healthcare site. Given prior echocardiogram AI models are trained on much less data (10-100x smaller training dataset sizes), we anticipated use of greater than 1 million samples would be sufficient to train a foundation model.
Data exclusions	Echocardiograms not classified as apical-4-chamber were excluded from the study.
Replication	95% confidence intervals were calculated using bootstrapping. The algorithm otherwise is deterministic and code is available.
Randomization	Patients were randomly divided into training, validation, and testing splits with approximate ratio of 89:1:10.
Blinding	This study is wholly retrospective and no additional human input was collected. Blinding was therefore neither possible nor necessary for this study.

Reporting for specific materials, systems and methods

We require information from authors about some types of materials, experimental systems and methods used in many studies. Here, indicate whether each material, system or method listed is relevant to your study. If you are not sure if a list item applies to your research, read the appropriate section before selecting a response.

Materials & experimental systems

n/a	Involved in the study
☒	☐ Antibodies
☒	☐ Eukaryotic cell lines
☒	☐ Palaeontology and archaeology
☒	☐ Animals and other organisms
☐	☒ Clinical data
☒	☐ Dual use research of concern
☒	☐ Plants

Methods

n/a	Involved in the study
☒	☐ ChIP-seq
☒	☐ Flow cytometry
☒	☐ MRI-based neuroimaging

Clinical data

Policy information about clinical studies
All manuscripts should comply with the ICMJE guidelines for publication of clinical research and a completed CONSORT checklist must be included with all submissions.

Clinical trial registration	Not a clinical trial
Study protocol	No prospective human subject activities were undertaken. Model design and training is described in the manuscript.
Data collection	The data was collected from the picture archival and communication system (PACS). This research was IRB approved.
Outcomes	The data was collected from the electronic healthcare record and structured reporting system. This research was IRB approved.

Plants

Seed stocks	*Report on the source of all seed stocks or other plant material used. If applicable, state the seed stock centre and catalogue number. If plant specimens were collected from the field, describe the collection location, date and sampling procedures.*
Novel plant genotypes	*Describe the methods by which all novel plant genotypes were produced. This includes those generated by transgenic approaches, gene editing, chemical/radiation-based mutagenesis and hybridization. For transgenic lines, describe the transformation method, the number of independent lines analyzed and the generation upon which experiments were performed. For gene-edited lines, describe the editor used, the endogenous sequence targeted for editing, the targeting guide RNA sequence (if applicable) and how the editor was applied.*
Authentication	*Describe any authentication procedures for each seed stock used or novel genotype generated. Describe any experiments used to assess the effect of a mutation and, where applicable, how potential secondary effects (e.g. second site T-DNA insertions, mosiacism, off-target gene editing) were examined.*
